# A longitudinal assessment of the serological response to *Theileria parva* and other tick-borne parasites from birth to one year in a cohort of indigenous calves in western Kenya

**DOI:** 10.1017/S003118201400050X

**Published:** 2014-05-16

**Authors:** H. KIARA, A. JENNINGS, B. M. DE C. BRONSVOORT, I. G. HANDEL, S. T. MWANGI, M. MBOLE-KARIUKI, I. CONRADIE VAN WYK, E. J. POOLE, O. HANOTTE, J. A. W. COETZER, M. E. J. WOOLHOUSE, P. G. TOYE

**Affiliations:** 1 The International Livestock Research Institute (ILRI), PO Box 30709, Nairobi–00100, Kenya; 2 The Royal Dick School of Veterinary Studies, University of Edinburgh, Easter Bush, EH25 9RG, UK; 3 The Roslin Institute, University of Edinburgh, Easter Bush, EH25 9RG, UK; 4 Centre for Immunity, Infection & Evolution, University of Edinburgh, EH9 3JT, UK; 5 School of Biology, University of Nottingham, Nottingham, NG7 2RD, UK; 6 Department of Veterinary Tropical Diseases, Faculty of Veterinary Science, University of Pretoria, Private Bag X04, Onderstepoort 0110, Republic of South Africa

**Keywords:** Cattle, serology, tick-borne diseases, haemoparasites, *Theileria parva*, longitudinal, Kenya

## Abstract

Tick-borne diseases are a major impediment to improved productivity of livestock in sub-Saharan Africa. Improved control of these diseases would be assisted by detailed epidemiological data. Here we used longitudinal, serological data to determine the patterns of exposure to *Theileria parva, Theileria mutans, Babesia bigemina* and *Anaplasma marginale* from 548 indigenous calves in western Kenya. The percentage of calves seropositive for the first three parasites declined from initial high levels due to maternal antibody until week 16, after which the percentage increased until the end of the study. In contrast, the percentage of calves seropositive for *T. mutans* increased from week 6 and reached a maximal level at week 16. Overall 423 (77%) calves seroconverted to *T. parva*, 451 (82%) to *T. mutans*, 195 (36%) to *B. bigemina* and 275 (50%) to *A. marginale. Theileria parva* antibody levels were sustained following infection, in contrast to those of the other three haemoparasites. Three times as many calves seroconverted to *T. mutans* before seroconverting to *T. parva*. No *T. parva* antibody response was detected in 25 calves that died of *T. parva* infection, suggesting that most deaths due to *T. parva* are the result of acute disease from primary exposure.

## INTRODUCTION

Tick-borne diseases are thought to constitute one of the major constraints to cattle productivity and the improvement of dairy industries in Africa (Perry and Young, 1995). Anaplasmosis, babesiosis and East Coast fever (ECF), caused by *Anaplasma marginale, Babesia bigemina* and *Theileria parva*, respectively, are three of the most important tick-borne diseases in eastern and central Africa (Uilenberg, 1995). Although the impact of these diseases has not been accurately and comprehensively quantified, it is generally believed that they cause enormous losses through morbidity, mortality, productivity losses and the cost of control (de Castro, 1997). In recent years, reduced funding for veterinary services following structural adjustment programmes in many developing countries and changes in production systems have greatly increased the impact of these diseases on livestock development (Cheneau *et al.* 2004).

Well-structured epidemiological studies on tick-borne diseases to estimate morbidity, mortality, case fatality rates and associated risk factors have been conducted in different production systems in eastern Africa (Gitau *et al*. 1997; Maloo *et al*. 2001; Muraguri *et al*. 2005; Okuthe and Buyu, 2006; Swai *et al*. 2007). The studies reported here are part of one such project: the Infectious Diseases of East Africa Livestock (IDEAL). The IDEAL project was a large, 3-year epidemiological study of calves in western Kenya aimed at alleviating the widely recognized lack of baseline epidemiological data on the dynamics and impacts of infectious diseases of cattle in eastern Africa.

We report here the patterns of exposure, as determined by serology, to infection with four of the most common tick-borne parasites in indigenous calves, from birth to one year. Three of the parasites (*T. parva, A. marginale* and *B. bigemina*) are widely believed to be pathogenic, while the fourth one, *Theileria mutans*, is mostly benign. The data presented here are essential in order to develop better, evidence-based management practices and control strategies to mitigate the effects of these infections and increase productivity in this important livestock system. The patterns of exposure which emerge from a longitudinal study also allow more accurate interpretation of cross-sectional epidemiological investigations.

## MATERIALS AND METHODS

### Study design

**Fig. 1.**
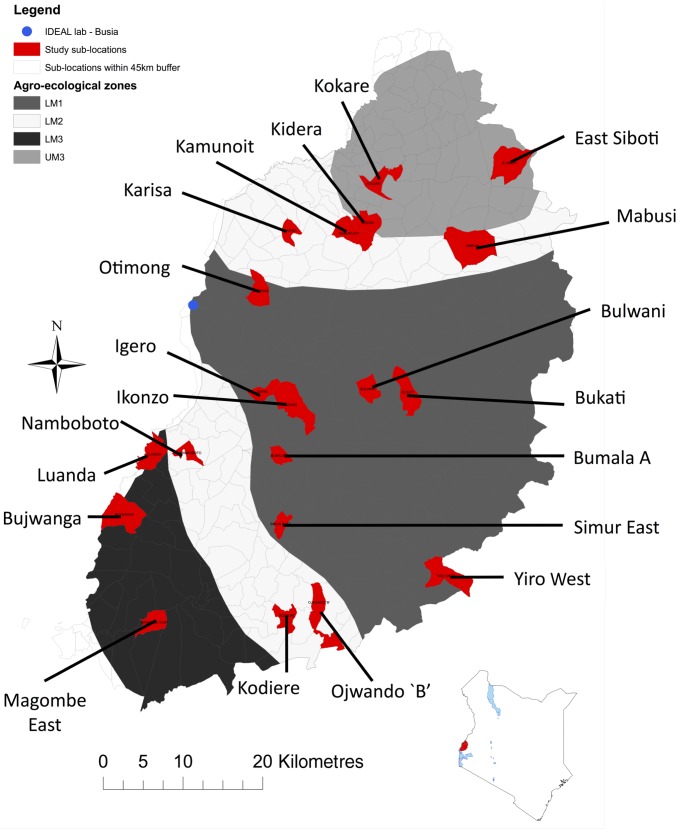
Map of the study area. A detailed map of the area in which the IDEAL study was undertaken is shown, together with an inset map showing the location of the study area in Kenya.

A detailed description of the IDEAL project has been provided previously (Bronsvoort *et al*. 2013). Briefly, the study was undertaken in an area of western Kenya extending from Mt. Elgon in the northeast to Lake Victoria in the southwest (Fig. 1). The area is characterized as a smallholder mixed crop/livestock production system with the small East African zebu as the predominant breed of cattle. The region has a warm and moist tropical climate with a bimodal rainfall pattern between March to May and October to December, although there is moderate rainfall throughout the year. Most of the area is cultivated but is interspersed with wetlands covered with grasslands that are often used for communal grazing. The IDEAL study was based in a field laboratory in Busia town on the Kenya/Uganda border. The study area is within a 45 km-radius semi-circle radiating from the field laboratory. It was stratified across four agro-ecological zones (AEZ): Lower Midland 1 (LM 1), Lower Midland 2 (LM 2), Lower Midland 3 (LM 3) and Upper Midland 3 (UM 3). AEZ describes the type of land and its suitability for different crops, and combines data on soil, topography and climate (Jaetzold and Schmidt, 1983). Using a stratified random cluster sampling (on AEZ) 20 sub-locations were selected. Sub-locations within each AEZ were selected using proportional random sampling with replacement. A sub-location is the smallest administrative unit of area in Kenya Government organization. No veterinary interventions were allowed, except on ethical grounds. If calves were treated for ethical reasons or inadvertently by the farmer, the observations from the treated calves were censored, in that observations at and subsequent to the treatment visit were not included in the analysis.

### Selection of calves

Up to 28 calves were randomly selected from each of the 20 sub-locations, such that a total of 548 calves were recruited into the study. All newborn calves in the sub-location were eligible for recruitment but recruited calves were required to pass a number of criteria for inclusion: (1) Calves had to have been born within 3 and 7 days of the date of recruitment; (2) they had to be born to a dam that had been on the farm for at least 1 year and not from artificial insemination; (3) the herd had to be extensively grazed; and (4) calves were only recruited from farms that did not already have a calf in the study at that time. To allow for seasonality in sampling calves were recruited from each sub-location every 5 weeks throughout the 2-year recruitment period.

### Sampling and laboratory analysis

Calves were visited at recruitment and routinely every 5 weeks for 1 year. Additional ‘clinical episode’ visits were undertaken if the calf was reported as exhibiting clinical signs, in particular pyrexia, enlarged lymph nodes and respiratory distress, among others (Bronsvoort *et al*. 2013). At each visit, an extensive clinical examination was carried out, quantitative outcome measures recorded and samples collected. Blood was collected in Vacutainer^™^ tubes (Becton Dickinson, England) from the jugular vein and stored in a chilled coolbox for transport back to the field laboratory. The blood was centrifuged at 12 000 ***g*** for 10 min. Sera were aspirated with sterile pipettes and stored in 1·8 mL Nunc cryovials at −40 °C before moving them to long-term storage at −80 °C until testing.

Species-specific antibody-capture ELISAs for *T. parva, T. mutans, A. marginale* and *B. bigemina* were carried out according to previously described methods (Katende *et al*. 1990, 1998; Morzaria *et al*. 1999; Tebele *et al*. 2000). For each sample, duplicate testing was carried out and a mean percentage positivity (PP) was calculated as the percentage ratio of the optical density (OD) obtained with the sample to the OD obtained with a strong positive control serum. The cut-off for the *T. parva* and *T. mutans* ELISA was ⩾20 PP, while for *B. bigemina* and *A. marginale* ELISA the cut-off was ⩾15 PP.

### Seroconversion rule

A seroconversion rule was employed that relied on PP results from two consecutive 5-weekly routine visits. For a calf to be defined as having seroconverted, the rule required that the PP value for the ‘seroconversion’ visit sample was greater than the cut-off value as defined above and greater than the previous visit (a rising titre). Under the rule, calves that were seropositive due to the presence of maternal antibodies were not classed as seroconverted, but calves which seroconverted during the period when maternal antibodies were present were identified.

### Cause of death

Full post-mortem examinations were carried out on calves that died. Gross abnormalities were noted and tissue samples were taken both into formalin and frozen for future analysis. Full histological examination was carried out on all available tissue. Most ECF cases were confirmed from macroscopic and microscopic examination of lung tissue, in addition to ante-mortem clinical signs and post-mortem observations of the animal. Final diagnoses of death were made by a committee of experts using all available diagnostic material. Where no diagnosis could be confirmed, the death was classified as due to unknown cause. A more detailed description of the mortality in the cohort is in Bronsvoort *et al*. (2013) and Thumbi *et al*. (2013).

### Data management and statistical analysis

The data collection and management have been previously described (Bronsvoort *et al*. 2013). All data extraction and analysis was conducted in R (R Development Core Team, 2010) using packages *RMySql* (James and DebRoy, 2012), *epicalc* (Chongsuvivatwong, 2010), *reshape* (Wickham, 2007), *ggplot2* (Wickham, 2009) and *survival* (Therneau and Lumley, 2010). The population-based estimates of seroprevalences for calves were determined using a weighted adjustment for the number of breeding dams in each sub-location and the s.e. was adjusted for clustering by sub-location using the R survey package (Lumley, 2004, 2012). The by-week baseline hazard (risk per unit time) of seroconversion to each parasite conditional on not having seroconverted previously was estimated using a time-discrete hazard model described by Singer and Willett (2003). This analysis was based on time to first occurrence of seroconversion, thus it only included observations up to and including the time of first seroconversion (if it occurred).

### Ethics statement

This project was approved by the University of Edinburgh Ethics Committee, the Kenyan Department of Veterinary Services and by ILRI's Institute Animal Care and Use Committee. Standard, peripheral venepuncture techniques were used to collect the blood samples. The calves were restrained by professional animal health assistants and veterinary surgeons, and a veterinary surgeon was available to examine any sick calf reported by recruited farmers. Any calves which were in severe distress due to trauma or disease were humanely euthanized by a veterinary surgeon. All farmers gave informed consent in their own language before recruitment of their calves began. The Ethical Review Committee of the University of Edinburgh (Animal (Scientific Procedures) Act, 1986) took into account the ethical issues enshrined in the Animals (Scientific Procedures) Act and approved the work (reference number OS 03-06).

## RESULTS

### Longitudinal assessment of the serological response to *T. parva, T. mutans, A. marginale* and *B. bigemina*

**Fig. 2.**
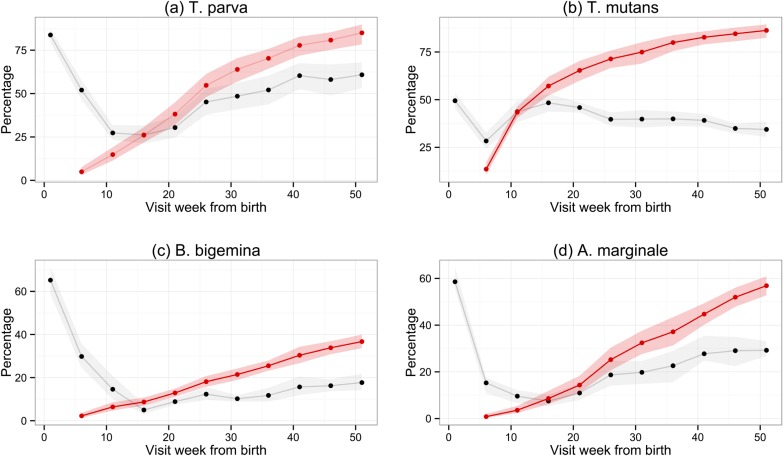
Comparison of the percentage of calves with positive antibody levels and the cumulative percentage of calves that had seroconverted at each routine visit. The black and red lines show, respectively, the estimated percentage of calves in the study site population whose PP result in ELISA was above the cut-off or cumulative percentage of calves that had seroconverted. The shaded areas represent the 95% confidence intervals.

Of the 548 calves recruited into the study, 455 survived for the study period (51 weeks), 88 died and five were removed from the study before 51 weeks (two were stolen, two were inadvertently treated with anthelmintics and one was treated on ethical grounds following injury). Sera from the routine 5-weekly visits for all calves were used to establish the longitudinal serological response in the population. Figure 2 shows the percentage of calves that were considered to be seropositive (sample PP value greater than the cut-off) at each visit. Following the recruitment visit, the percentage of calves seropositive for each haemoparasite decreased, presumably due to removal of maternally derived antibody from the calves’ circulation. For *T. parva, A. marginale* and *B. bigemina*, this decrease continued until week 16, after which the percentage of seropositive calves showed a general increase up to and including the final visit at 1 year (week 51). For *T. mutans*, the trend in seropositivity differed from that of the other parasites, as it reached its lowest level at week 6 and increased rapidly until week 16 (the visit where the percentage of seropositive animals for all other species was lowest). After week 16, the percentage of *T. mutans*-seropositive calves decreased gradually towards the final visit at 1 year.

The use of seropositivity based on a simple cut-off value to determine the exposure to haemoparasites in the calf population is confounded by the presence of maternal antibody. To avoid this, we devised a seroconversion protocol based on the results of two consecutive visits (Methods). The seroconversion results for each parasite are also shown in Fig. 2, which indicates the cumulative proportion of calves in the population that had seroconverted by each routine 5-weekly visit. The general pattern is an increase in the number of animals that seroconverted, which began from about weeks 6 to 16 and diminished towards the end of the study period. In all, we observed that 423 calves (77%) seroconverted to *T. parva*, 451 (82%) to *T. mutans*, 195 (36%) to *B. bigemina* and 275 (50%) to *A. marginale.* When these raw numbers are adjusted for deaths and censoring and also for the different population sizes in each sub-location, the estimates (with 95% confidence intervals) for cumulative seroconversion at 51 weeks are 85·9% (78·4–89·7) for *T. parva*, 86·3% (82·4–89·5) for *T. mutans*, 36·7% (33·6–39·9) for *B. bigemina* and 56·9% (52·8–60·9) for *A. marginale*.

**Fig. 3.**
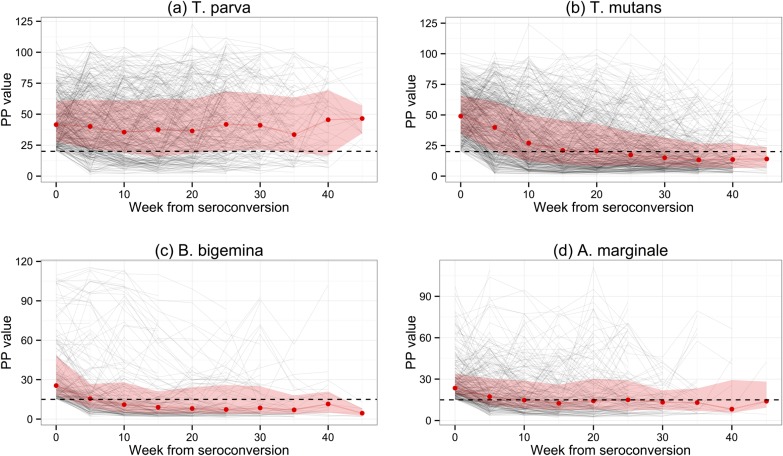
Levels of serum antibodies to the four haemoparasites in those calves that had seroconverted by each routine visit. The red line represents the estimated median PP value obtained in ELISA for those calves in the population at the respective 5-weekly visits at or following seroconversion. The black background lines are the plots of individual calves. The shaded areas represent the 95% confidence intervals.

When the lines indicating the proportion of calves that were seropositive are compared with those for seroconversion, the presence of maternal antibody in calf serum is clearly evident. It is also evident that, from between week 11 to week 16 onwards, the percentage of seropositive animals fell below the percentage that had seroconverted. This is especially evident for *T. mutans*, and is presumably due to a decrease in the antibody titre over time in some infected animals. This was investigated further by examining the antibody levels in only those calves that had seroconverted. Figure 3 shows the median PP values for each haemoparasite, and also the PP values for individual calves, at each week following seroconversion. Except for *T. parva*, there was a gradual decrease in the median PP value to at or below the cut-off value, indicating that most calves eventually become seronegative following exposure. In contrast, the median PP value for *T. parva* was sustained following initial exposure, although a substantial number of calves also became seronegative. There was a particularly rapid decrease in antibody levels for *T. mutans*, which is consistent with the decrease in the percentage of seropositive animals described above (Fig. 2b). The 50% interquartile range at each time point following seroconversion shown in Fig. 3 demonstrates the considerable variation in the PP values obtained with sera from individual animals in the population.

### Infection pressure exerted by the four haemoparasites

**Fig. 4.**
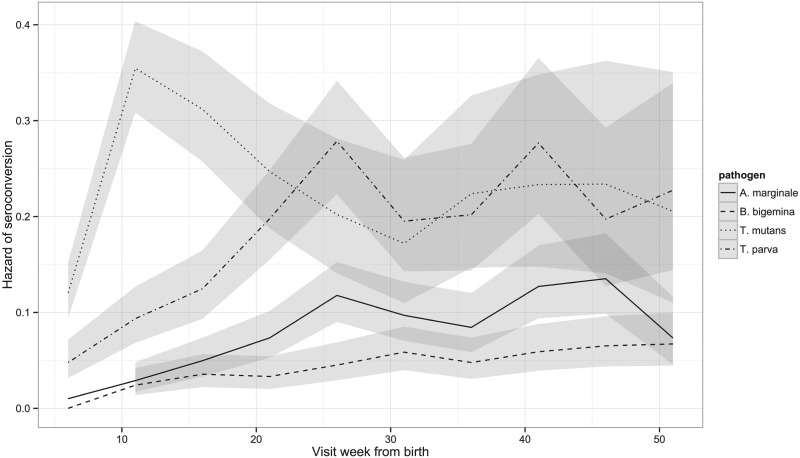
Hazard of seroconversion to *T. parva, T. mutans, B. bigemina* and *A. marginale* by routine visit. The lines represent the estimated hazard of seroconversion for *T. parva, T. mutans, B. bigemina* and *A. marginale*, as indicated. The shading represents the 95% confidence intervals.

Although the percentage of calves that had seroconverted to *T. parva* and to *T. mutans* by 1 year is about the same, Fig. 2 shows that seroconversion to *T. mutans* generally occurs before that to *T. parva*. This was confirmed on calculating the median age to seroconversion, which is 107 days for *T. mutans* and 178 days for *T. parva*. It is also evident when the hazard of seroconversion is plotted through time (Fig. 4). The maximal hazard for *T. mutans* occurred at week 11, whereas that for *T. parva* was observed at week 26. The hazard for both gradually declined after these peaks, apart from a small secondary increase for *T. parva* at week 41. By comparison, the hazard of seroconversion to *A. marginale* and *B. bigemina* increased gradually over the year, reflecting a much lower infection pressure.

**Fig. 5.**
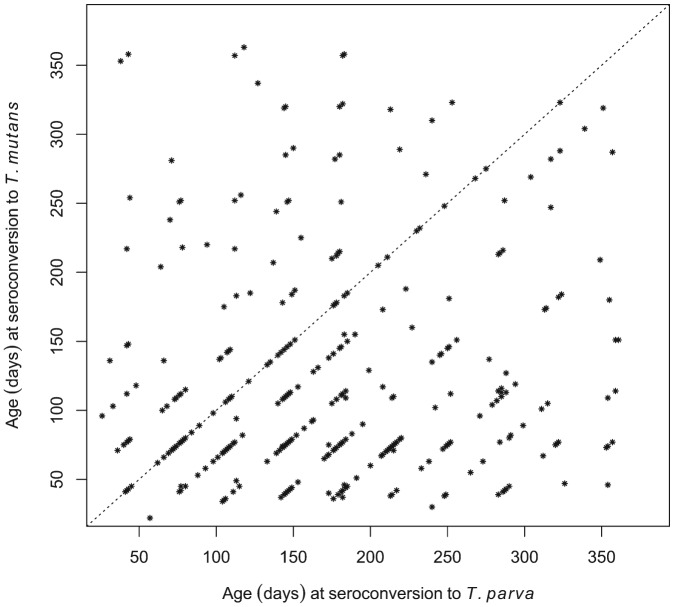
Comparison of the ages at which individual calves seroconverted to *T. parva* and to *T. mutans*. The dot plot shows the respective ages of the calves at the routine visit at which they were deemed to have seroconverted to the haemoparasites.

Although seroconversion to *T. mutans* generally preceded that to *T. parva* at the population level, we also wished to establish if this was consistently the case in individual calves. Of the 380 calves that seroconverted to both *T. mutans* and *T. parva*, 245 were recorded as having seroconverted to *T. mutans* first, 79 calves to *T. parva* first, and 56 simultaneously to both (seroconversion occurred during the same 5-week inter-visit period). This is illustrated in Fig. 5, where most of the data points are below the diagonal identity line. Figure 5 also illustrates that in some calves there was a considerable interval, about 300 days in some cases, between the two seroconversion events.

### Serological responses in calves that died of *T. parva* infection during the study

Clinical, laboratory and post-mortem reports of the 88 animals that died during the 51-week study period were examined by an expert veterinary panel to establish the likely cause of death of these animals. ECF, which results from infection with *T. parva*, was considered the primary cause of death in 32 cases and a contributing cause in 2 animals, as reported previously (Bronsvoort *et al*. 2013; Thumbi *et al*. 2013). (Three of the 34 animals were euthanized and are considered here as dying of the primary pathology seen post-mortem.) A key question in the epidemiology of the disease is whether calves die from an initial exposure to *T. parva*, or experience several infections before succumbing. In other words, does an initial exposure usually generate a sufficient immunological response to protect calves from subsequent field exposure? To address this, we examined the serological profiles of the ECF cases to see if any of these animals had previously seroconverted.

**Fig. 6.**
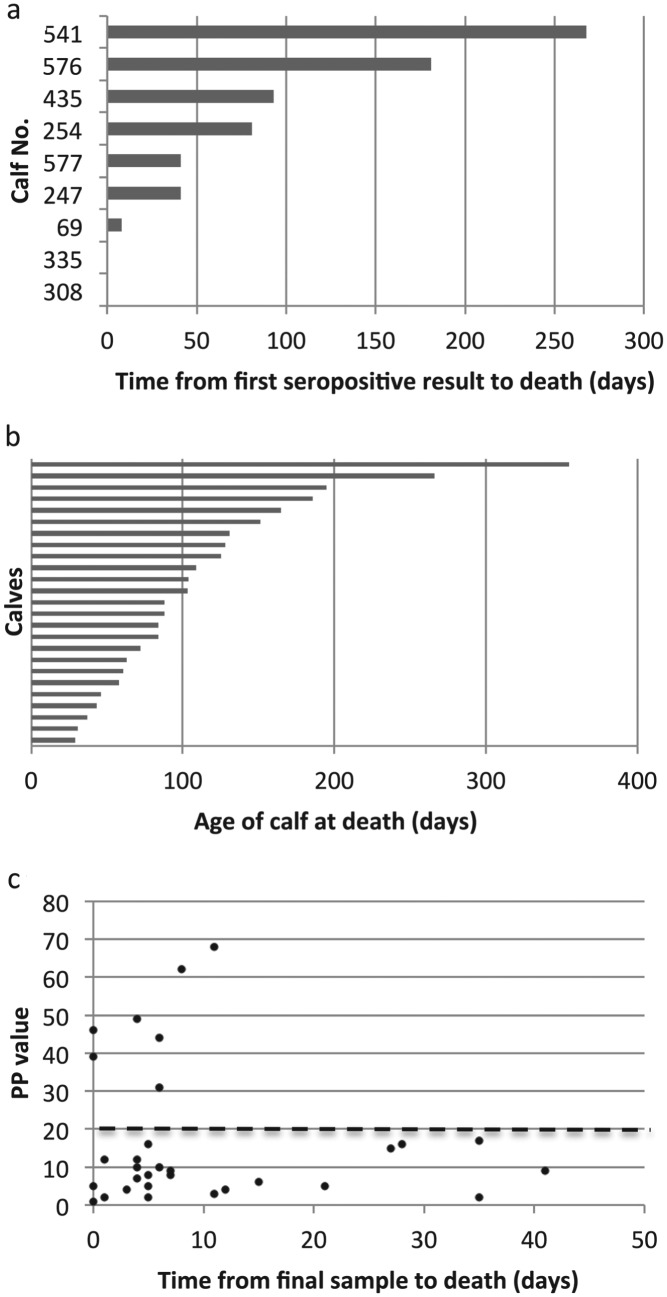
Serological responses to *T. parva* in calves that died from ECF. (a) The time from the first seropositive result to death is shown for the nine calves that were seropositive on at least one occasion. Each horizontal bar represents an individual animal and the labels on the vertical axis indicate the individual calf identification number. Calves 308 and 335 died on the day of sampling, so no horizontal bar appears for these animals. (b) The age at death of 25 seronegative calves that appeared to die from ECF following a single exposure to *T. parva*. Each horizontal bar represents an individual animal. (c) The mean PP value obtained in the *T. parva* ELISA from the final sample from calves that died of ECF is plotted against the time between the date of sampling and the death of the calf. The results from three calves that died when maternal antibody appeared still to be present are not included. The dashed line represents the cut-off value for seropositivity.

Of the 34 fatal cases of *T. parva* infection, only seven seroconverted before death according to the seroconversion rule. One of these animals had a single seropositive result, which occurred on the final routine visit. When the serology results from non-routine, ‘clinical episode’ visits close to the time of death were included, a further two animals were identified which had a single seropositive result, also on the final visit. (A further three animals were positive by ELISA but this was attributed to the presence of colostral antibody.) Figure 6a shows how long the seropositive calves survived following their first seropositive result. It can be seen that only six calves appear to have had a previous infection with *T. parva*, as they seroconverted from between 41 and 268 days before death. The antibody response in the other three animals could be attributed to the fatal infection. Thus, the lack of a sustained antibody response in most of the calves suggests that calves that die from ECF do so as a result of their first infection with *T. parva*.

Figure 6b shows the ages at death of the 25 calves that appeared to die without developing an antibody response to *T. parva*. The ages at death of these calves ranged from 29 to 355 days, with a median age at death of 88 days. This is considerably less than the median age at seroconversion for the population as indicated above, and suggests that younger calves are more likely to die following infection with *T. parva*. Interestingly, four of these calves survived for over 180 days before dying.

The results indicate that an anti-*T. parva* antibody response is not a general feature of calves that die of ECF. It could be argued that in some instances there was time between the final visit and the death of the calf for an antibody response to have occurred. This was examined by plotting the PP value of the final visit sample and the time between the final visit and death. Figure 6c shows the results for all calves, except the three calves that died when maternal antibody appeared to be present at the final visit. Only seven calves were seropositive at the visit closest to death, as the PP value from two calves that had previously seroconverted had declined to below the cut-off point by the time of death. It can be seen that, although six of the remaining 24 calves died at least 3 weeks after their final sample was obtained, 15 seronegative calves died within 7 days of final sampling, including one calf that had previously seroconverted. The lack of an antibody response in these calves argues against the use of serology as a diagnostic tool to confirm death due to ECF.

## DISCUSSION

The longitudinal serological analysis in the IDEAL calf population revealed a consistent and logical pattern for the antibody responses to three of the haemoparasites examined. For all parasites, there was a substantial proportion of seropositive animals at recruitment, presumably due to maternal antibody. This is supported by the fact that 99% of the farmers in the study reported that the calves received colostrum (Toye *et al*. 2013). Thereafter, the proportion of seropositive animals for *T. parva, A. marginale* and *B. bigemina* decreased from birth until week 16, followed by a general increase until the end of the study, presumably due to the generation of endogenous antibody following exposure to the parasites. This agrees with our earlier study on colostral uptake, where very few calves had detectable maternal antibody after 16 weeks (Toye *et al*. 2013).

The exception to the general pattern of serological responses was seen with the generally benign parasite, *T. mutans.* Two differences were observed. First, the initial decline in the percentage of seropositive animals was much more short-lived, reaching its lowest level in week 6 rather than week 16. This indicates earlier generation of endogenous antibody presumably due to a higher infection pressure for *T. mutans* leading to exposure in very young calves. This is also reflected in the earlier increase in the number of animals that seroconverted to *T. mutans* compared with the other parasites. Of particular interest was the comparison between seroconversion to *T. parva* and *T. mutans*. Although the percentage of calves that seroconverted to the two parasites in the first year of life was similar (77% and 82%, respectively), the mean age at seroconversion to *T. mutans* was much earlier (107 compared with 178 days), and over three times as many calves seroconverted first to *T. mutans* than to *T. parva*. Similar observations were made in two previous studies in the Trans-Mara district of Kenya, in which the antibody response to *T. mutans* considerably preceded that to *T. parva* (Moll and Lohding, 1984, 1986). For example, in the first study, the mean age at active response to *T. mutans* was estimated to be 70·4 days compared with that of *T. parva* at 110·4 days. Intriguingly, these researchers recorded many more *Rhipicephalus appendiculatus* ticks than *Amblyomma* spp., the tick vectors of *T. parva* and *T. mutans*, respectively. Although comprehensive tick counts were not undertaken in the IDEAL study, many more animals were seen to carry *R. appendiculatus* than *Amblyomma* spp. ticks. Although this appears to be counterintuitive to the notion of higher infection pressure from *T. mutans*, the previous report (Moll and Lohding, 1984) also noted much higher parasite infection rates in *Amblyomma* spp. ticks and suggested that this was the cause of the earlier seroconversion to *T. mutans*. An alternative explanation is that maternal antibody inhibits the induction of antibody production following infection with *T. parva* but not, or to a lesser extent, with *T. mutans*. This inhibitory effect has been seen with various antigens and it has been observed that the effect occurs to different extents with different antigens (Hodgins *et al*. 2004; Pastoret, 2007). Interestingly, a recent study on a single farm in Uganda, in which reverse line blot was used to detect parasite DNA, found that the average age at first infection with *T. parva* was 53 days, whereas with *T. mutans* it was 74 days (Asiimwe *et al*. 2013).

The second difference observed with the serological response to *T. mutans* was that the number of seropositive animals slowly declined after a peak at week 16. In contrast, the number of animals seropositive to the other parasites generally increased until the end of the study. Nevertheless, a common observation for all four parasites was that the percentage of calves seropositive at the routine visits towards the end of the study period was less than the cumulative percentage that had seroconverted by that visit. With one exception, this is presumably due to a general decline in antibody levels after exposure. It appears that the general decrease in antibody levels following seroconversion is not boosted by re-infections, suggesting the development of immunity in infected animals. The exception was the case of *T. parva*, where the median PP value was generally sustained for up to 46 weeks following infection. Although, as discussed below, the data presented here suggest that immunity does develop in *T. parva*-infected animals in the study area, secondary, non-lethal infections may occur which boost the antibody responses in these animals.

Similar to other studies in the region, the proportion of calves seropositive to *A. marginale* and *B. bigemina* was significantly lower than those seropositive to *T. parva* and *T. mutans* (Okuthe and Buyu, 2006; Swai *et al*. 2007). It is not clear if this was due to a lower abundance of *Rhipicephalus* (*Boophilus*) *decoloratus*, the main tick vector of *A. marginale* and *B. bigemina* in the study area, because as discussed earlier detailed tick counts were not conducted in this study. It could however also be due to a lower capacity of these ticks to transmit parasites compared with *R. appendiculatus* and *Ambylomma* spp., the main field vectors of *T. parva* and *T. mutans*, respectively.

No antibody response to *T. parva* was seen in 25 of the 34 calves that died of ECF. The 25 calves included 14 animals that died within 7 days of their last sampling. Thus, seroconversion is not typically seen in calves that die of ECF, which suggests that antibody-detection assays are of limited value in diagnosing severe cases of the disease. This field observation is in line with the lack of antibody response in cattle challenged by lethal inoculation with *T. parva* stabilates in pen trials (Toye *et al.* unpublished results). It extends the previous observation of Thumbi *et al*. (2013) that seropositivity to *T. parva* was associated with a reduced risk of death. Our study indicates that this is due to the fact that only animals that survive *T. parva* infection produce detectable antibodies, rather than there being a survival advantage conferred by anti-*T. parva* antibodies. It is presumably a consequence of death occurring before the normal development of the antibody response, possibly compounded by the disruption to normal lymphoid function due to the proliferation of infected lymphocytes in lymphoid organs.

Of the remaining nine calves, six seroconverted at least 41 days prior to death. As the time from infection to detectable antibody response is 13–28 days (Katende *et al*. 1998) and the time from infection to death is similar (Ndungu *et al*. 2005), these results suggest that these six animals survived an initial *T. parva* infection, only to succumb to a second infection, presumably antigenically distinct from the first, or to a recrudescence of the primary infection perhaps due to exposure to another pathogen. In comparison, most calves in the cohort seroconverted to and survived infection with *T. parva* (Thumbi *et al*. 2013), suggesting that immunity to field challenge develops after a single infection in most calves in this population. This is somewhat surprising, as immunity to *T. parva* is strain-specific (Taracha *et al*. 1995) and antigenic heterogeneity is a common feature of *T. parva* parasites, particularly in parasites derived from buffalo (Pelle *et al*. 2011). It may be that the parasites circulating in the study region have limited antigenic diversity due possibly to the absence of buffalo in the region, or that most calves in this population survive initial infections through an innate resistance which allows them to develop a broad immunity to subsequent challenge. It should also be noted that, as 25 calves were infected with *T. parva* but did not develop detectable antibodies, the percentage of calves reported as having seroconverted slightly underestimates the number of calves exposed to the parasite.

In summary, the seroconversion data indicate that calves in this farming system can be infected with tick-borne pathogens from the first few weeks of life, suggesting that any preventative measures for these diseases must be administered early. The serological response pattern showed similar dynamics for three of the haemoparasites. The exception was *T. mutans*, where seroconversion occurred much earlier and the antibody response was less sustained compared with the other haemoparasites. It would be interesting to explore if this is linked to the lower virulence associated with *T. mutans* compared with the other organisms. The general lack of seroconversion in those animals that die of *T. parva* infection suggests that antibody-based diagnosis of fatally infected calves is of little clinical use. On the other hand, as almost all of the calves which survive their initial exposure to *T. parva* appear to survive subsequent challenges, less stringent measures may be required to prevent ECF in seropositive animals.
